# Gastric plexiform fibromyxoma: a case report and literature review

**DOI:** 10.3389/fonc.2025.1686973

**Published:** 2025-11-20

**Authors:** Li Zheng, Jin Wang

**Affiliations:** Department of Pathology, The Second People’s Hospital of Hefei, Hefei, China

**Keywords:** plexiform fibromyxoma, mesenchymal tumours, stomach, immunohistochemistry, benign tumor

## Abstract

Plexiform fibromyxoma (PF) is a rare mesenchymal tumour that primarily occurs in the stomach, with the antrum and pyloric region being the most common sites. A few cases have also been reported in the duodenum, jejunum, mediastinum, gallbladder, and other locations. Over 100 cases have been reported in the literature, with more than 30 cases reported in our country. A rare case of PF occurring in the cardia and fundus of the stomach is reported in this study. The tumour tissue showed a multinodular, plexiform growth pattern between the muscle bundles of the gastric wall, in which a myxoid matrix and thin-walled vessels were visible. The tumour cells were spindle shaped or short spindle shaped, with a mild change in morphological appearance, and mitotic figures were rare. The tumour cells showed immunohistochemical expression of vimentin and SMA, with focal expression of calponin and CD10. The Ki-67 proliferation index was approximately 5%. The plexiform fibromyxoma was characterized by benign biological behaviour, with surgical excision as the primary therapy. The patient was followed up for about 2 years after surgery without any tumour recurrence or metastasis.

## Introduction

Plexiform fibromyxoma is a recently recognized gastrointestinal mesenchymal tumour. It is also referred to as a plexiform angiomyxoid myofibroblastic tumour (PAMT) because it involves tumour cells with myofibroblastic differentiation and a stroma rich in thin-walled small vessels and a myxoid matrix. It was first reported by Takahashi et al. in 2007. In 2010, PF was officially listed in the WHO Classification of Tumours of the Digestive System as a type of gastric mesenchymal tumour. The tumour primarily occurs in the stomach, with the most common locations being the antrum and pyloric region, followed by the gastric body, fundus, and cardia. A few cases have also been reported in the esophagus, duodenum, jejunum, mediastinum, gallbladder, and other locations (1–5). Genetic mutations in the glioblastoma-associated oncogene homolog 1 (GLI1) and the long non-coding RNA metastasis-associated lung adenocarcinoma transcript 1 (MALAT1) have been reported in a subset of PF cases (6). In other instances, polyploidy of the 12q13 locus harboring GLI1 has been observed, leading to overexpression of the GLI1 protein (7). Moreover, recent studies documented a case of PF characterized by co-amplification of GLI1, CDK4, and MDM2, accompanied by TP53 mutations (8). PF exhibits benign biological behaviour and should be differentiated from other gastrointestinal spindle cell tumors. In this work, we reported a rare case of PF, together with describing the clinical characteristics, histopathologic and immunophenotypical features, as well as the discussion on the misleading differential diagnosis, to raise the awareness of this enigmatic neoplasm and help avoid potential diagnostic pitfalls.

## Case presentation

**Figure 1:**
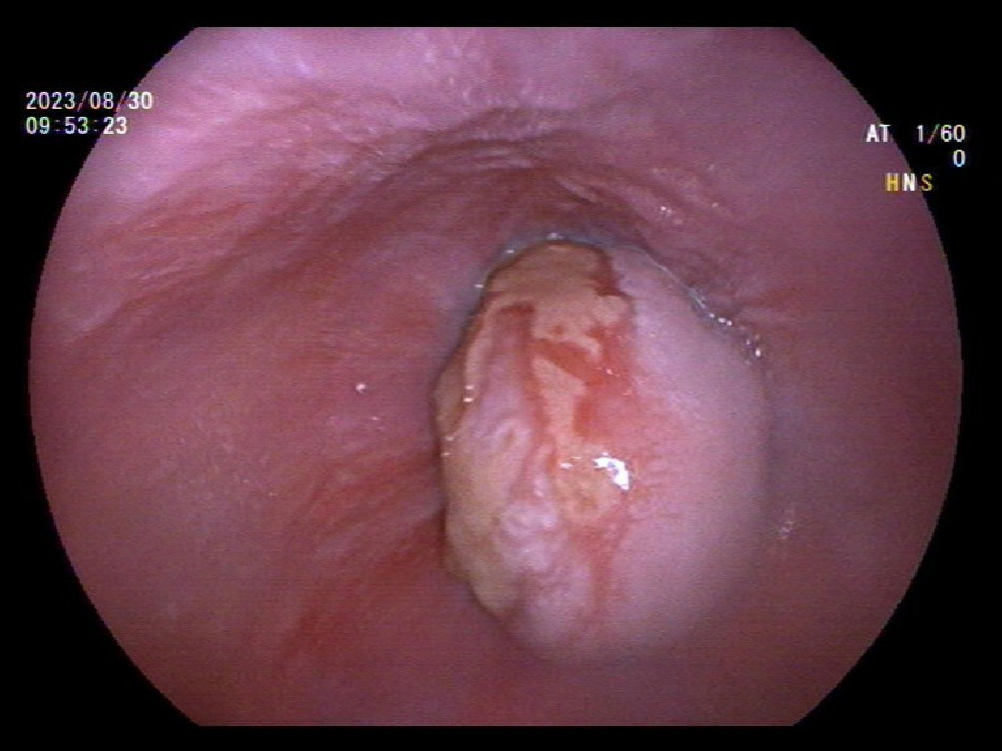
Gastroscope shows an endophytic mass within gastric fundus with mucosal ulcer.

**Figure 2:**
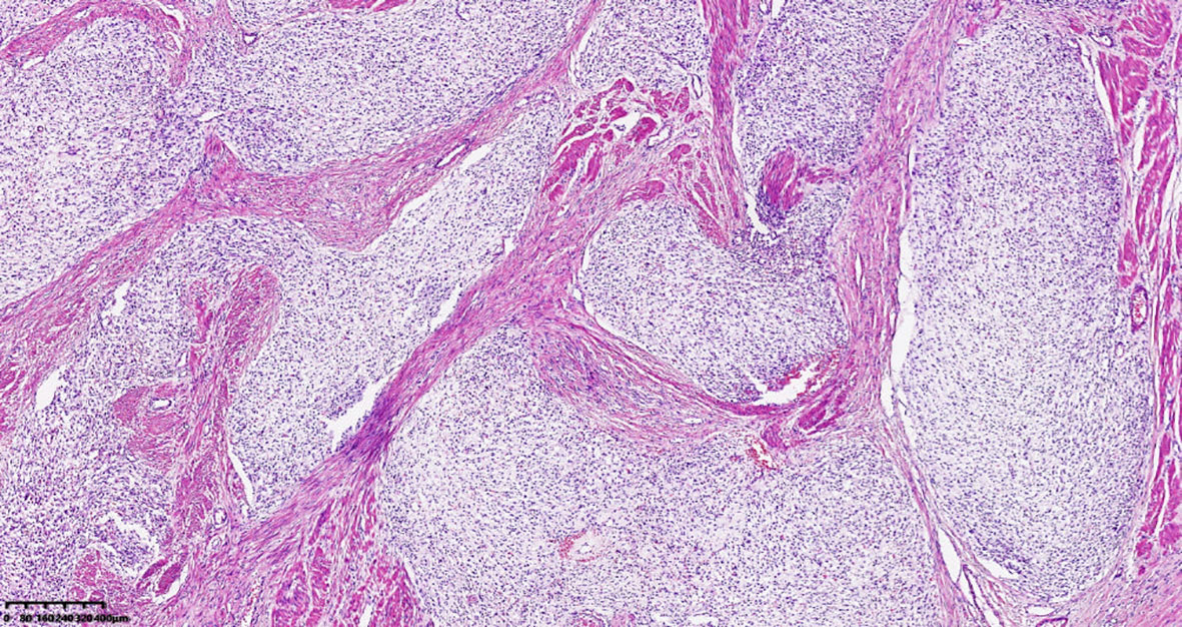
Plexiform fibromyxoma shows characteristic plexiform growth. HE, 40×.

**Figure 3:**
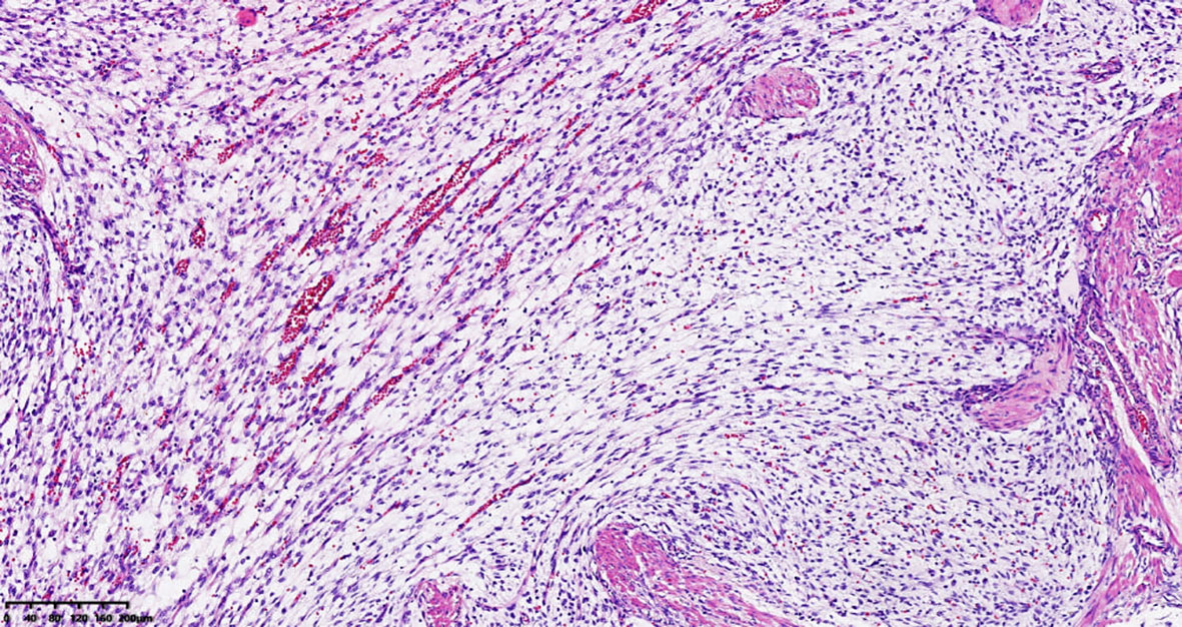
The tumor is composed of bland spindle to ovoid cells. HE, 100 ×.

**Figure 4:**
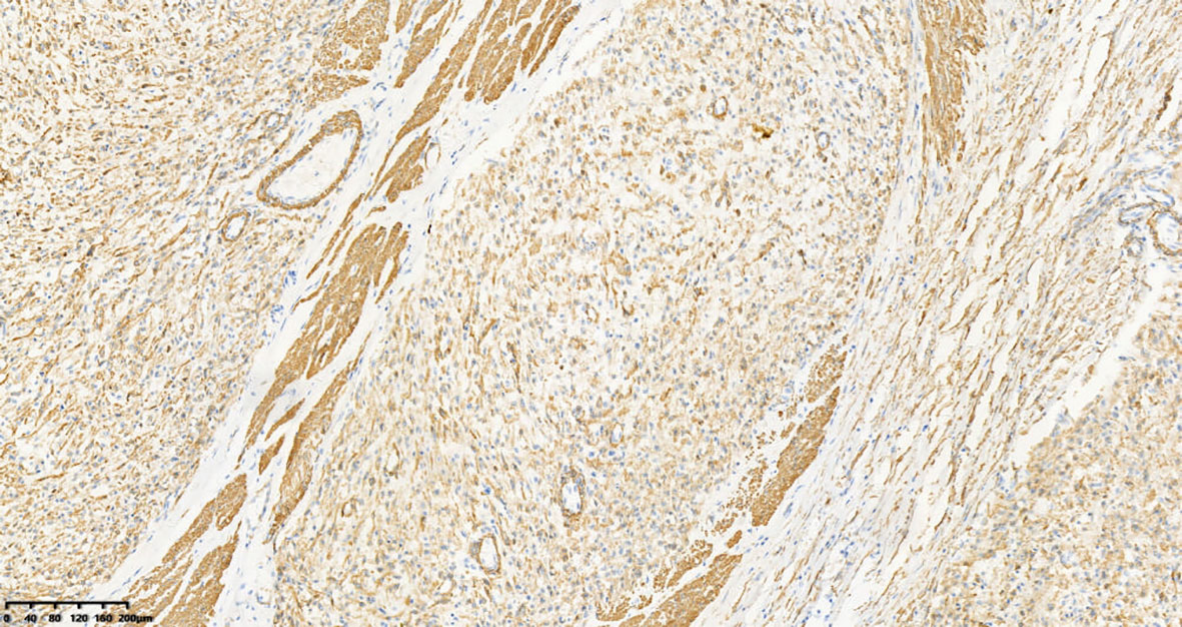
The tumor cells are positive for SMA. IHC, 100×.

A 39-year-old male with a history of hypertension, smoking, and alcohol consumption, was admitted to the hospital with a diagnosis of “upper gastrointestinal bleeding” due to melena accompanied by haematemesis for more than 2 hours. After admission, gastroscopy revealed a large submucosal elevated tumour on the posterior wall of the greater curvature of the fundus, with an irregular surface fester accompanied by the formation of a scab, extending upwards to involve the cardia (**Figure 1**). Owing to failure of endoscopic resection and a tendency for bleeding, the patient underwent laparoscopic partial gastrectomy under general anaesthesia. Intraoperative findings revealed that the tumour was located at the cardia and fundus of the stomach, crossed the dentate line, and had an intragastric growth pattern. Gross examination revealed grey–white nodular tissue measuring 7 cm × 4.5 cm × 2.5 cm, with a grey-white and tough cut surface. Additionally, a piece of mucosal tissue measuring 3.5 cm × 1.5 cm × 0.5 cm was observed. Histologically, the tumour tissue had a multinodular, plexiform growth pattern between the muscle bundles of the gastric wall, in which a myxoid matrix and thin-walled vessels are visible. (**Figure 2**). The tumour cells were spindle shaped or short spindle shaped, with a mild change in morphological appearance, and mitotic figures were rare (**Figure 3**). Immunohistochemistry revealed that the tumour cells were positive for vimentin and SMA expression (**Figure 4**), focal expression of calponin and CD10, and weak expression of β-catenin, with no expression of CD117, DOG-1, desmin, caldesmon, HMB45, ALK, CD34, S-100, CK, or SOX10. The Ki-67 proliferative index was approximately 5%. The final pathological diagnosis was gastric plexiform fibromyxoma.

## Discussion

Plexiform fibromyxoma is extremely rare, with only over 100 cases reported in the literature, of which more than 30 cases were reported in our country. PF occurs across a wide age range, from 5 to 81 years, with a predominant occurrence in middle-aged individuals, and there is no significant sex difference. The clinical features of PF are nonspecific and often include upper gastrointestinal symptoms, such as abdominal pain, distension, discomfort, haematemesis, and melena, as well as rare perforation (9). In this case, the tumour occurred at the cardia and fundus, and the patient sought medical attention because of gastrointestinal bleeding. On the basis of imaging and endoscopic examination, PF typically presents as a submucosal tumour or polypoid protuberance, with a smooth mucosal surface or ulceration. This tumour is difficult to differentiate macroscopically from other submucosal tumours, such as gastrointestinal stromal tumours (GISTs), schwannomas, and leiomyomas. A definitive diagnosis requires histopathological examination.

Plexiform fibromyxoma typically originates in the submucosa or muscularis propria. When the size of the tumour is large, it can extend beyond the serosal layer. The tumour diameter ranges from 0.8 cm to 19 cm, and it generally appears as multiple nodular, mucinous, or gelatinous masses. It may be accompanied by bleeding or cystic degeneration. Its cut surface is grey–white or grey–red. The histological morphology of the tumour shows a characteristic nodular and plexiform growth pattern, interspersing between the smooth muscle bundles of the gastric wall. The tumour cells are short, spindle shaped or oval and located within a myxoid, fibromyxoid, or collagenic matrix. The matrix may contain numerous branched, thin-walled capillaries. The cell morphology has a mild change, with a pale and eosinophilic cytoplasm. The cell boundaries are unclear, the nuclear chromatin is delicate, the nucleolus is not obvious, and mitotic figures are rare. There is no tumour necrosis. Ultrastructural examination shows that the tumour exhibits myofibroblastic differentiation. Immunohistochemistry typically reveals diffuse expression of vimentin and SMA; focal expression of desmin, caldesmon, and CD10; and no expression of CD34, S100, CD117, DOG1, ALK, or EMA. The myxoid matrix shows positive staining with Alcian blue. In addition, some studies have reported that PF may show focal expression of CK (10), so local keratin staining cannot exclude PF. Diagnosing PF through preoperative and intraoperative biopsy specimens and/or fine needle aspiration (FNA) is challenging. Without the aid of immunohistochemistry, misdiagnosis is highly likely, which may result in overtreatment. Lai J et al. reported that 6 out of 7 PF patients were misdiagnosed with GISTs through preoperative endoscopic ultrasound-guided fine needle aspiration or intraoperative frozen sections, and one of them also received Gleevec therapy (11).

The diagnosis of Plexiform fibromyxoma needs to be differentiated from that of other spindle cell tumours of the stomach. (i) GIST, the most common mesenchymal tumour in the digestive tract, must be considered first and ruled out when diagnosing PF. Tumours may be located in the submucosa, muscularis propria or subserosa. Morphology includes the spindle cell type, epithelial cell type, and mixed cell type, among which spindle cell type is the most common. The spindle cell type of GIST shows spindle-shaped and short spindle-shaped tumour cells that are arranged in a bundle-shaped, woven-shaped, or vortex-shaped pattern, with varying cell densities and mostly without significant polymorphisms. Vacuolation may be observed at the nuclear ends. The stroma may be accompanied by hyalinization and collagenization, with significant myxoid degeneration in some of the framework. A very small number of gastric GIST subgroups may exhibit a plexiform growth pattern, particularly in paediatric-type and SDH-deficient GISTs (12). Tumour cells may immunohistochemically express CD117 and/or DOG-1, and genetic mutations in C-KIT or PDGFRα may be present, which helps differentiate PF from GIST. (ii) Schwannoma accounts for approximately 2% - 6% of gastrointestinal mesenchymal tumours. Schwannomas are typically located in the submucosal or muscularis propria layers, with well-defined borders and no true fibrous capsule. They are composed of spindle-shaped or oval-shaped Schwann cells. Varying amounts of collagen fibres can be observed between tumour cells. The classic type exhibits alternating patterns of dense, bundle-like areas (Antoni A areas) and loose, reticular areas (Antoni B areas). The most prominent feature of gastrointestinal schwannomas is the presence of sheets of mature lymphocytes surrounding the tumour cells, known as lymphocytic cuffing. Immunohistochemical detection of the expression of S-100, SOX10, and GFAP in tumour cells can assist in differential diagnosis. (iii) Leiomyoma is a benign mesenchymal tumour with smooth muscle cell differentiation that is most commonly found in the oesophagus within the digestive system, whereas it is rare in the gastrointestinal tract. The tumour cells are spindle shaped, with a mild change in morphology and no atypia. They are arranged in bundles, interlaced patterns, or whorled formations. Immunohistochemically, in addition to being positive for SMA, these cells also express desmin and caldesmon, which helps differentiate them from PF. (iv) Inflammatory myofibroblastoma (IMT) is a myofibroblastic tumour with intermediate differentiation potential. When it occurs in the gastrointestinal tract, it can involve the submucosa, the muscularis propria, and the mesentery. The tumour cells are spindle shaped with vacuolated nuclei, which may be accompanied by small nucleoli. The cells may vary in density and may present with a loose, mucinous background. The tumour is often associated with infiltration by inflammatory cells such as lymphocytes, plasma cells, and eosinophils. Immunohistochemically, the IMT cells commonly express SMA and desmin, with approximately 60% showing positive ALK expression. A small portion may exhibit CK expression. Molecular testing reveals ALK gene rearrangement and, in some cases, ROS1 gene rearrangement, which helps differentiate it from PF. (v) Inflammatory fibrous polyps are polypoid lesions that occur in the gastrointestinal tract. The tumour is located mainly in the submucosa, but it may involve the mucosa, and in a small number of cases, it can affect the muscularis propria or entire layers. The tumour is composed of spindle-shaped and short-spindle cells arranged in bundles or interlacing patterns. There is a proliferation of small blood vessels in the stroma. Tumour cells may form an onion-skin-like structure around thin-walled blood vessels, accompanied by significant chronic inflammatory cell infiltration. The tumour does not exhibit a plexiform growth pattern; immunohistochemical studies have detected the expression of CD34 but not of SMA.

The molecular genetic characteristics of Plexiform fibromyxoma are not yet fully understood. The patient in this case has no family history of the disease. Some studies have reported the presence of MALAT1-GLI1 gene fusion in PF, which can lead to overexpression of the Gli1 protein. However, the specificity, biological consequences, and exact mechanisms of tumorigenesis associated with this genetic alteration in PF remain to be further investigated. In addition, mutations in the C-KIT and PDGFRA genes have been negative in all reported cases of PF. This genetic analysis, which is helpful for differential diagnosis, confirms the distinct genetic features of PF compared with those of GIST (13).

According to the current literature, Plexiform fibromyxoma exhibits benign biological behaviour. The primary therapy is surgical excision, which mainly involves distal or partial gastrectomy. There have been no reports of recurrence or distant metastasis after complete tumour excision. However, reports on PF are limited, and some studies have indicated the presence of vascular invasion (14). Therefore, the benign nature of PF still requires longitudinal observation, and studies on more cases are needed. Our patient was followed up for about 2 years after surgery without any tumour recurrence or metastasis.

In summary, Plexiform fibromyxoma is a rare mesenchymal tumour that primarily occurs in the stomach. The diagnosis mainly depends on histological and immunohistochemical findings. At present, there are few reports on PF, so more cases and follow-up observation are needed to assist in the correct diagnosis of PF and to better understand the biological characteristics of this tumor.

## Data Availability

The original contributions presented in the study are included in the article/supplementary material. Further inquiries can be directed to the corresponding author.
